# Mountain rock glaciers contain globally significant water stores

**DOI:** 10.1038/s41598-018-21244-w

**Published:** 2018-02-12

**Authors:** D. B. Jones, S. Harrison, K. Anderson, R. A. Betts

**Affiliations:** 1 College of Life and Environmental Sciences, University of Exeter, Penryn Campus, Penryn, Cornwall, TR10 9EZ UK; 2 Environment and Sustainability Institute, University of Exeter, Penryn Campus, Penryn, Cornwall, TR10 9EZ UK; 3 College of Life and Environmental Sciences, University of Exeter, Streatham Campus, Exeter, EX4 4QE UK; 4 Met Office, FitzRoy Road, Exeter, Devon, EX1 3PB UK

**Keywords:** Cryospheric science, Hydrology

## Abstract

Glacier- and snowpack-derived meltwaters are threatened by climate change. Features such as rock glaciers (RGs) are climatically more resilient than glaciers and potentially contain hydrologically valuable ice volumes. However, while the distribution and hydrological significance of glaciers is well studied, RGs have received comparatively little attention. Here, we present the first near-global RG database (RGDB) through an analysis of current inventories and this contains >51,000 RGs. Using the RGDB, we identify key data-deficient regions as research priorities (e.g., Central Asia). We provide the first approximation of near-global RG water volume equivalent and this is 62.02 ± 12.40 Gt. Excluding the Antarctic and Subantarctic, Greenland Periphery, and regions lacking data, we estimate a near-global RG to glacier water volume equivalent ratio of 1:618. Significant RG water stores occur in arid and semi-arid regions (e.g., South Asia East, 1:57). These results represent a first-order approximation. Uncertainty in the water storage estimates includes errors within the RGDB, inherent flaws in the meta-analysis methodology, and RG thickness estimation. Here, only errors associated with the assumption of RG ice content are quantified and overall uncertainty is likely larger than that quantified. We suggest that RG water stores will become increasingly important under future climate warming.

## Introduction

In semi-arid and arid high mountain systems glaciers and seasonal snowpack form natural buffers to hydrological seasonality, as seasonal meltwater contributions smooth the effects of highly variable summer precipitation and associated irregular runoff^1–3^. Described as the world’s natural “water towers”^4^, glacier- and snowpack-derived meltwater are critical to ecological, social and economic systems in these regions. Additionally, mountain water stores provide buffering capacity for surrounding lowlands^5^. Elevation dependent warming (i.e. an amplified rate of warming with altitude) suggests that high-altitude environments will likely experience comparatively faster warming than lower altitude areas^6^. Furthermore, high-altitude hydrological resources are highly sensitive to environmental change^3,7^. Indeed, between 2003 to 2009 glacier volume loss globally was estimated to be −259 ± 28 Gt yr^−1^ ^8^. With projected atmospheric warming, long-term glacier and seasonal snowpack changes are expected to impact significantly hydrological resources stored within high mountain systems^9^. Small and low-lying glaciers are particularly likely to be sensitive to warming, with many disappearing^10–12^. In the short-term glacier shrinkage results in increased runoff. However, following “peak non-renewable water”^13^, summer runoff will significantly reduce in semi-arid and arid regions^14,15^. Additionally, a warming-induced precipitation shift from snowfall to rainfall^16^ combined with a temporal shift towards earlier snowpack melt^17^, will further lead to runoff reduction.

**Figure 1:**
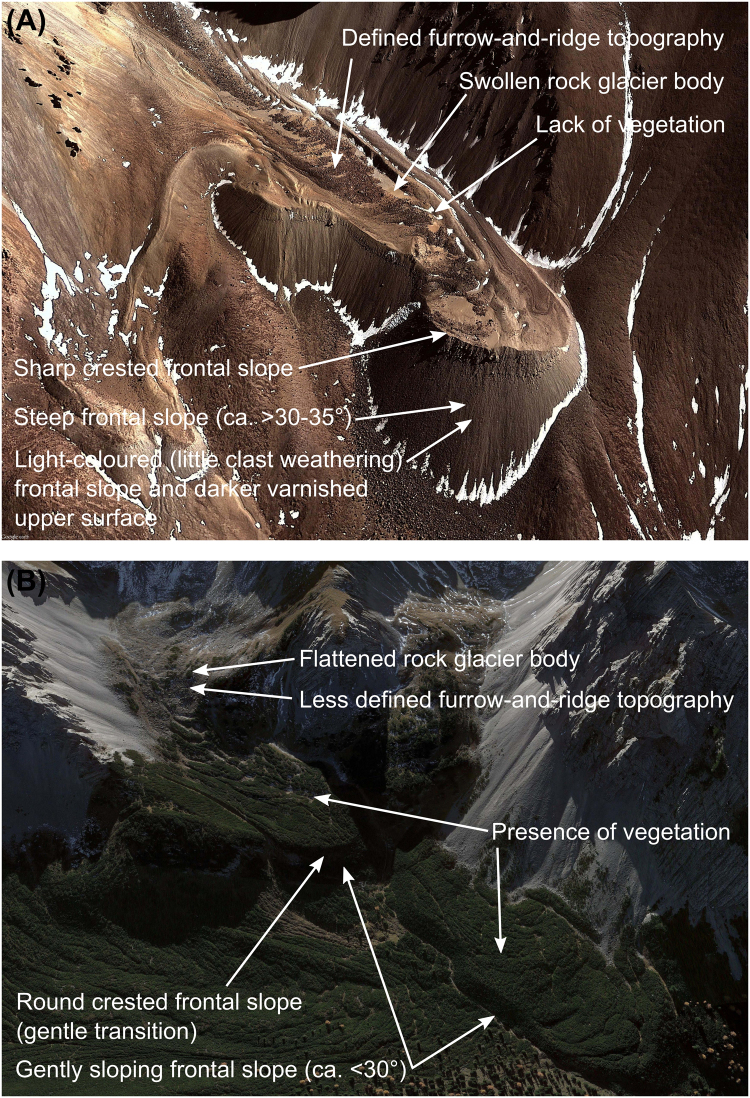
Annotated examples of RGs: (**A**) the intact Caquella RG Bolivia (21°29′S, 67°55′W). Image data: Google Earth (version 7.1.5.1557, Google Inc., California, USA), DigitalGlobe; imagery date: 20 July 2010; and (**B**) a relict RG complex, Nördliche Kalkalpen (Northern Calcareous Alps), Austria (47°19′N, 11°23′E). Image data: Google Earth, DigitalGlobe; imagery date: 17 October 2017.

Consequently, effective water resource management in terms of climate change adaptation strategies is critical. However, this is hampered by an incomplete understanding of all components of the hydrological cycle in high mountain systems. Whilst much has been written on the hydrological role of glaciers^18^, that of rock glaciers (RGs) has received comparatively little attention^19^. RGs are cryospheric landforms that are formed by gravity-driven creep of accumulations of rock debris supersaturated with ice. They are characterised by a seasonally frozen, clastic-blocky surficial layer ~0.5 to 5 m thick that thaws each summer (this is known as the *active layer*)^20^. RGs are described as active or inactive if they contain ice beneath the active layer. These are described collectively as intact RGs. Those containing no or minimal ice content are termed relict RGs^21^. RGs are thermally decoupled from external micro- and meso-climates because of the insulating effect of the active layer, which is shown to slow the rate of ice melt within RGs^20^. Consequently, RGs respond to climate change at comparatively longer time scales than glaciers^22^. Therefore, RGs are more climatically resilient than glaciers and form frozen water stores of potentially significant hydrological value^23^. Indeed, under future climate warming RGs are expected to form a larger component of base flow to rivers and streams^24^. RGs containing ice generally display slow movement rates (mm yr^−1^ to cm yr^−1^) because of the gravity-driven creep of the ice-supersaturated RG body. This movement creates distinctive morphometric characteristics that enable feature identification and activity classification (i.e. intact or relict) (Fig. 1).

Ubiquitously distributed through the world’s high mountain systems^25^, RGs have been found in greater numbers than glaciers in certain regions, for example the central Andes^24^. Despite this, RGs are not currently included in global-scale glacier databases such as the Global Land Ice Measurements from Space (GLIMS) glacier inventory. To date RG distribution has only been researched at regional scales (e.g., European Alps, PermaNET^26^). Although described as the “most pressing need” in RG research, as yet no global-scale RG inventory exists^27^. This prevents full assessment of RG distribution and their hydrological value as water stores, and forms the motivation for this study.

## Brief Methods

As the primary objective of this study, we present the first near-global-scale RG database (RGDB). The RGDB is shared as a Microsoft Excel database available in the supplementary information online. We argue that data dissemination forms a positive step towards scientific transparency and open-access research, benefitting both the scientific and local/regional communities. The RGDB was developed through meta-analysis of systematic inventory studies published prior to October 2017 (see Methods). In this study, ‘systematic inventory studies’ refer to the strategic and complete mapping of RG features within a study area. We identified these using ISI Web of Science, Scopus, ProQuest Dissertations and Theses, Google Scholar, and National Snow and Ice Data Center (NSIDC) search tools (Supplementary Table S1). The meta-analysis resulted in 131 systematic inventory studies. To avoid duplicate data (i.e. overlapping study areas) some studies were excluded or partially excluded. Consequently, 76 studies form the RGDB.

Within this study, our secondary objective was to establish the relative hydrological contributions of glaciers and RGs at a near-global scale. Therefore, it is important to be able to compare quantitatively the estimated water volume equivalent of RGs vs. glaciers. With regards to RGs, thickness-area (T-A) scaling relations, i.e. *H* = *cA*^*β*^ where mean RG thickness (H) is calculated as a function of surface area (A) and two scaling parameters (c and β), were applied. The scaling parameters applied here were derived from the empirical rule established by Brenning^28^ (see equation [1] in Methods). RG volume was estimated through multiplication of (H) and (A). Where complete RG inventories were available, RG surface area data were extracted for each individual feature for use in the T-A relationship. A three-step approach to determine RG volume was used where inventory data was incomplete or unknown (see Methods; Supplementary Fig. S1). By definition, RGs do not contain 100% ice (i.e. ice content is spatially heterogeneous), but because few geophysical investigations of RGs have been conducted, precisely estimating ice content is difficult. Here, we assumed ice content to be 40–60% by volume, enabling calculation of lower (40%), mean (50%), and upper (60%) estimates, following other studies^29–31^. Subsequently, water volume equivalent was calculated assuming an ice density conversion factor of 900 kg m^−3^. With regards to glaciers, the Randolph Glacier Inventory version 4.0 (herein RGIv4.0) released December 2014^32^ “provides a globally complete set of outlines for all [ice- and debris-covered-] glaciers outside the two ice sheets Greenland and Antarctica”^33^. For each glacier of the RGIv4.0, Huss and Hock^34^ calculated glacier volume and ice thickness distribution through the application of an ice-thickness distributed model^35^ (see Methods). These global-scale data were used within this study. To better enable regional assessment, the RGDB was divided into RGIv4.0 adapted regions (see Methods; Supplementary Fig. S2 and Supplementary Table S2). The above-described approaches have potentially large associated uncertainties as described in the ‘study uncertainty’ section. Therefore, the volumetric results presented here represent a first-order approximation. Nevertheless, we thought it prudent to include these results, as there exists a need to make significant advances in this research field in the context of continued climate change.

## Results and Discussion

### RGDB meta-analysis results

Searches of the ISI Web of Science, Scopus and ProQuest Dissertations and Theses generated 799, 1023, and 359 studies respectively (Supplementary Table S1). Excluding duplicates, peer-reviewed studies (i.e. ISI Web of Science and Scopus) published within the previous decade (2007–2017) totalled 579. In total, 131 systematic RG inventory studies resulted from the categorisation of the available collated literature, of which ~72% were published post-2000 and ~63% during the last 10 years (2007–2017). This reflects, as in previous studies^36^, an increased interest in RG research in the last decade. After duplicate RG data, i.e. overlapping study areas, were excluded, 76 studies formed the final RGDB. Systematic RG inventories forming the RGDB were predominantly generated using expert photomorphic mapping from remote sensing image data, with landforms manually identified and digitised based upon geomorphic indicators (see Fig. 1). Recent technological advancements in remote sensing science have:

(i) provided the opportunity for large-scale geomorphological surveys. For instance, fine spatial satellite image data are accessible freely through Google Earth, including SPOT and DigitalGlobe (e.g., QuickBird, Worldview-1 and 2, and IKONOS). The RGDB includes studies that have used the Google Earth platform to compile systematic RG inventories^37,38^;
(ii) provided open-access to <1 m resolution airborne laser scanning (LiDAR) data, enabling relict RGs covered by dense vegetation to be systematically mapped^39^. This is important as relict landforms strongly influence catchment hydrology^40^; and
(iii) provided accessible interferometric synthetic aperture radar (InSAR) data (e.g., ESA Sentinel-1). In the context of this study, InSAR has enabled systematic RG mapping through the investigation of surficial kinematics (i.e. feature movement). Subsequently, activity status classification can be defined with greater accuracy (e.g. Liu *et al*.^41^).

### RGDB coverage

Our results from the meta-analysis suggest that the number of systematic RG inventory studies is relatively strongly, and significantly related to the total number of RGs identified (*r* = 0.71, *p-value* = < 0.01). Study density is highest in Central Europe (*n* = 27), followed by South America (*n* = 17) and North America (*n* = 7) (Table 1). These RGI regions account for ~67% of systematic RG studies within the final RGDB. Therefore, we can use the RGDB to identify regions that have been the focus of far less scientific research. Significant gaps in the available data are evident. For example, no systematic RG inventory studies have been compiled for Arctic Canada North, Arctic Canada South, Russian Arctic, and South Asia West RGI regions. Furthermore, only ~9% of studies contained within the RGDB cover the Hindu Kush Himalayan range (Central Asia = 5, South Asia East = 2, South Asia West = 0), home to the largest ice volume outside of the polar regions and a region that the authors, amongst others have found contains thousands of RGs^37,38,42^. The RGDB includes only data derived from the meta-analysis, with overall coverage determined by available systematic RG inventory studies. Given the above-mentioned knowledge gaps, the RGDB presented here represents a ‘near-global’ resource.

**Table 1:** RGDB data reflecting total studies, RG numbers, areas, and water volume equivalents at the regional (RGI regions) and near-global scale.

RGI region	No. studies (*n*)	Rock glaciers (*n*)			Rock glacier area			WVEQ
		Total	Intact	Relict	Total	Intact	Relict	
	(−)	(−)	(−)	(−)	(km^2^)	(km^2^)	(km^2^)	(Gt)
Antarctic and Subantarctic	3	35	33	2	3.21	3.01	0.20	0.04 ± 0.01
Caucasus and Middle East	3	983	551	432	113.22	68.52	44.70	0.93 ± 0.19
Central Asia	5	2187	1991	196	291.61	290.35	17.24	4.08 ± 0.82
Central Europe	27	11968	4189	7728	752.28	326.18	495.84	3.13 ± 0.63
Greenland Periphery	1	390	186	204	46.00	21.91	24.09	0.30 ± 0.06
Iceland	2	181	121	60	61.37	47.57	13.80	0.82 ± 0.16
New Zealand	1	386	166	220	42.56	20.47	22.09	0.28 ± 0.06
North America	7	13833	7874	5959	1710.67	1131.08	628.73	15.57 ± 3.11
North Asia	5	7207	3431	3776	801.51	422.77	378.73	5.74 ± 1.15
Scandinavia	2	248	67	181	26.44	8.26	18.18	0.11 ± 0.02
South America	17	6991	4579	2412	976.51	725.01	251.66	11.14 ± 2.23
South Asia East	2	6513	4356	2157	1417.57	950.80	466.77	19.48 ± 3.90
Svalbard and Jan Mayen	1	500	238	262	55.66	29.36	26.30	0.40 ± 0.08
**NEAR-GLOBAL**	**76**	**51422**	**27783**	**23588**	**6298.62**	**4045.30**	**2388.33**	**62.02** ± **12.40**

The water volume equivalent calculations are associated with an estimated range of ice content by volume (%), with lower (40%), mean (50%), and upper (60%) estimates. Those RGI regions where no systematic RG inventory studies have been undertaken (i.e. Arctic Canada North, Arctic Canada South, Russian Arctic, South Asia West, and Svalbard and Jan Mayen) are excluded from the table. See Supplementary Fig. S1 and Supplementary Table S2 for details on the RGI regions. Values are reported to two decimal places.

WVEQ = Water volume equivalent.

### Glacier- and rock glacier- hydrological value

The RGDB presented here contains 51,422 RGs (intact = 27,783, relict = 23,588) covering an estimated area of ~6,300 km^2^. From this, we present a first-order approximation of volumetric ice content contained within intact RGs. In total, we estimate that intact RGs contain a total ice volume of ~69 Gt assuming 50% ice content by volume. Therefore, intact RGs contain a total water volume equivalent of between 49.61 and 74.42 Gt, equivalent to ~54–81 trillion litres (Table 1), if a possible range of ice content between 40% and 60% is considered. Regionally, intact RGs located within South Asia East (19.48 ± 3.90 Gt), North America (15.57 ± 3.11 Gt) and South America (11.14 ± 2.23 Gt) likely contain the largest water stores. Conversely, water volume equivalents found within the Antarctic and Subantarctic, Greenland Periphery, New Zealand, and Scandinavia RGI regions are the smallest, with the upper estimate (i.e. 60% ice content by volume) containing <0.88 Gt combined. Importantly, long-term RG water stores in arid and semi-arid regions are large (e.g., South America = 11.14 ± 2.23 Gt). This is particularly significant where glacial meltwater provides an important portion of potable water, for example in La Paz, Bolivia where future water scarcity due to the pressures of climate change, poor infrastructure and increasing population is likely^43,44^.

The RGIv4.0 contains 197,654 digital glacier outlines covering an area of ~726,792 km^2^ globally^34^. Furthermore, Huss and Hock^34^ estimated glaciers to contain 138,074 Gt of ice, equating to an estimated water volume equivalent of 124,266 Gt (Table 2). As a result, the total ratio of RG to glacier water volume equivalent is estimated to be 1:2,226 (Table 2). This implies that glaciers contain a store of water 2,226 larger than that of RGs at the near-global scale.

**Table 2:** RG and glacier total areas and water volume equivalents at the regional (RGI regions) and near-global scale.

RGI region	Rock glacier		Glacier		Ratio
	Area	WVEQ	Area	WVEQ	Rock glacier: glacier WVEQ
	(km^2^)	(Gt)	(km^2^)	(Gt)	
Antarctic and Subantarctic	3.21	0.04	132,867.00	39,834.76	1:995,869
Arctic Canada North	No Data	No Data	104,873.00	24,742.59	∞
Arctic Canada South	No Data	No Data	40,894.00	7,272.89	∞
Caucasus and Middle East	113.22	0.93	1,139.00	55.38	1:60
Central Asia	291.61	4.08	62,606.00	3,688.13	1:904
Central Europe	752.28	3.13	2,063.00	103.37	1:33
Greenland Periphery	46.00	0.30	89,721.00	13,958.78	1:46,529
Iceland	61.37	0.82	11,060.00	3,001.45	1:3,660
New Zealand	42.56	0.28	1,162.00	55.38	1:198
North America	1710.67	15.57	101,274.00	17,628.45	1:1,132
North Asia	801.51	5.74	3,430.00	147.67	1:26
Russian Arctic	No Data	No Data	51,592.00	11,326.51	∞
Scandinavia	26.44	0.11	2,851.00	132.91	1:1,208
South America	976.51	11.14	31,679.00	4,873.21	1:437
South Asia East	1417.57	19.48	21,799.00	1,103.85	1:57
South Asia West	No Data	No Data	33,859.00	2,791.02	∞
Svalbard and Jan Mayen	55.66	0.40	33,922.00	7,357.80	1:18,395
**NEAR-GLOBAL**	**6298.62**	**62.02**	**726,792.00**	**138,074.14**	**1:2,226**

The ratios of RG to glacier water volume equivalence is also given. RG water volume equivalent uses the average ice content by volume (50%). Values are reported to two decimal places.

Excluding those RGI regions where no systematic RG inventory studies have been undertaken (i.e. Arctic Canada North, Arctic Canada South, Russian Arctic, and South Asia West), the estimated ratio of RG to glacier water volume equivalence is 1:1,482. For completeness we also excluded the Antarctic and Subantarctic and Greenland Periphery RGI regions, similar to other studies^45^, along with the aforementioned RGI regions where no systematic RG inventories have been undertaken. The resulting estimated ratio of RG to glacier water volume equivalence globally is 1:618. The ratio of RG to glacier water volume equivalence varies greatly geographically, between 1:26 (North Asia) and 1:18,395 (Svalbard and Jan Mayen), excluding the Antarctic and Subantarctic and Greenland Periphery RGI regions. However, continental-extent ratios are not reflective of national-level or regional-level ratios. For example, RG to glacier water volume equivalence ratios of 3:1 (semi-arid Chilean Andes [29°–32°])^46^ and 1:3 (West region, Nepal)^38^ have been reported.

A number of RGI regions are underrepresented within the RGDB (Table 2). Importantly, this includes RGI regions within which severe water stress will likely result from future climate warming, for example, those RGI regions encompassing the Hindu Kush Himalaya (Central Asia, South Asia East, South Asia West). From this we argue that the RGDB data set likely underestimates the potential hydrological value of RGs as future water stores. Furthermore, with continued climate-driven deglaciation, high mountain systems are in the initial stages of transitioning from glacial- to paraglacial-dominated process regimes^47^. The term *paraglacial* is defined as “…nonglacial earth-surface processes, sediment accumulations, landforms, landsystems and landscapes that are directly conditioned by glaciation and deglaciation”^48,49^. Modification of rock slopes (through rock slope failures [RSFs]) dominate the rock slope paraglacial system, as high mountain systems respond to deglacial unloading or debuttressing following the exposure of glacially steepened rockwalls by glacier downwastage and retreat^48^. This may subsequently increase glacier surface insulation through enhanced debris-supply. Therefore, frozen water store preservation may occur, as glaciers transition to RG forms^50^. Sasaki *et al*.^51^ estimated global supraglacial debris-cover to be ~43,750 km^2^ (~20,830 km^2^ classified as thick) and also provided regional estimations. For example, significant debris-cover in Central Asia (13,965 km^2^), South Asia East (5,555 km^2^), and South Asia East (3,343 km^2^) suggests that potentially ‘suitable habitats’ for RG development exist within these locations. We suggest, therefore, that these are priority regions for future systematic RG inventory studies. Furthermore, given the global glacier volume loss projections of 25–48% between 2010–2100^34^, we further suggest that RGs will play an increasingly important future role in regional water supply.

### Study uncertainty

It is important to acknowledge possible sources of uncertainty within this study, particularly given the necessity to generalise with regards to RG ice volume and the associated water volume equivalent calculations. Possible sources of uncertainty are discussed below.

1. *Inherited errors within the RGDB:* Whereas automated and semi-automated techniques have enabled the mapping and monitoring of clean-ice glaciers from optical satellite data^52^, these approaches are generally unsuitable for mapping debris-covered glaciers (e.g., Alifu *et al*.^53^). This is because both supraglacial-debris (upon the glacier) and debris at the glacier margins share a common source, and thus spectral similarity of features “render them mutually indistinguishable”^52^. This limitation is also applicable to RGs (e.g., Brenning^54^). Therefore, manual RG identification and digitisation using geomorphic indicators (Fig. 1) remains the optimal approach for inventory compilation. This approach is used by many studies included within the RGDB. However, this methodology is inherently subjective and introduces potential uncertainties (see Scotti *et al*.^55^ and Jones *et al*.^38^). Furthermore, Whalley *et al*.^56^ have previously highlighted the problem of mapping ‘hidden’ ice with respect to RGs. The RGDB presented here includes only meta-analysis derived data from the available systematic RG inventory studies. As such, any errors present (and where those errors are quantified or unquantified) in the original studies will be present here.
2. *The meta-analysis methodology:* The RGDB was developed through meta-analysis of systematic RG inventory studies published prior to October 2017, using ISI Web of Science, Scopus, ProQuest Dissertations and Theses and NSIDC search tools. Therefore, the RGDB comprehensiveness is predominantly governed by the availability of openly accessible academic information on the topic. As such, studies outside of these research-bounds, in particular those published in non-ISI indexed journals, may have been missed. Furthermore, integration of systematic RG inventory data into the RGDB was hampered by: (i) non-standardised inventory datasets (see Cremonese *et al*.^26^); (ii) non-English language writing; (iii) the absence of an accessible open-access database (only 43 of studies in the full RGDB [~33%] had linked databases); and (iv) incomplete inventory data. With regards to (ii), we used Microsoft Translator/PROMT Translator to increase the accessibility of non-English manuscripts, and thus increased the completeness of the RGDB. Studies where Microsoft Translator/PROMT Translator was used are noted as such within the RGDB files available in the supplementary information. Additionally, a possible source of error occurs in situations where systematic RG inventory data are either incomplete or unknown ([iii] and [iv]). As previously mentioned, in these situations we chose to implement a three-step approach to determine RG activity status, area, and ice volume (Supplementary Fig. S2). This three-step approach has potentially large associated uncertainties as we have, of necessity, to generalise.
3. *Methodology for determining (a) glacier- and (b) rock glacier-hydrological stores:* Regarding (a), within this study results provided in Huss and Hock^34^ were adopted. For each glacier of the RGIv4.0 Huss and Hock^34^ calculated glacier volume and ice-thickness distribution through the application of their model (herein HF-model), updating the previous results of Huss and Farinotti^35^ that relied upon RGIv2.0 data (released June 2012). “[T]he RGI is intended to be a snapshot of the world’s glaciers as they were near the beginning of the 21^st^ century (although in fact its range of dates is still substantial)”^57^. Indeed, within the RGIv4.0 released in December 2014^32^, the average satellite acquisition date (±1 standard deviation) of inventoried glacier outlines within each of the 19 first-order regions ranges from 1970 ± 19 (North Asia) to 2009 ± 2 (Alaska)^34^. Given that glacier volume loss globally was estimated to be −259 ± 28 Gt yr^−1^ between 2003 to 2009^8^, RGI-derived data may overestimate glacier area. With regards to the HF-model, previously uncertainty assessments have been undertaken, the results of which have been summarised and discussed in detail^35,58^. Lastly regarding (a), results within Huss and Hock^34^ were presented as sea-level equivalent (SLE) assuming an ice density of 900 km m^−3^ and an ocean area of 3.625 × 10^8^ km^2^. For the purposes of this study, these results were converted from SLE to ice volume for each RGI first-order region. Converted ice volumes may slightly differ compared to the original dataset, as Huss and Hock^34^ reported SLE only to 2 decimal places.

With regards to (b), we acknowledge that the results presented here represent a first-order approximation. Although “[a] detailed examination of the surface features of a RG [as used in many studies included in the RGDB] may also give a general indication of the position, activity, and quantity of hidden ice”^56^, generally, RG thickness and average ice content are unknown variables. Direct measurements of internal structure are limited, due to the practicalities of field-based research (e.g., direct drilling, geophysical investigations) in largely remote locations^27^. Indeed, regarding the paucity of such scientific investigations, it has been purported that “[m]embers of the mining community have had more opportunities to study RGs internally than have geomorphologists”^59^. Furthermore, unless *in situ* internal structure data are spatially distributed with good coverage of the entire feature extent, the ice-thickness and ice-debris ratio at any location remains an assumption^60^. Therefore, here T-A scaling relations, i.e. *H* = *cA*^*β*^ (see Methods), were applied. Scaling parameters derived from the empirical rule established by Brenning^28^ were used (equation [1]). According to this power-law relationship, a RG sized 0.01 km^2^ and 1 km^2^ would contain an ice-debris layer 20 m and 50 m thick, respectively. This estimation of RG thickness is based upon morphometric field measurements^28^. However, this T-A scaling relationship was developed for RGs in Central Chile. As such, this approach cannot account for regional specificities of RGs around the globe, and thus we cannot be certain of the suitability of our approach to RGs globally. As an alternate approach, it can be argued that a thickness of >~20 m is necessary for active RGs to creep^29,61^. Indeed, some previous studies have adopted a consistent RG permafrost thickness of 20 m for all RG sites^62^. However, Burger *et al*.^63^ (cf. Table 4) and Janke *et al*.^27^ detail examples where quantitative field measurements indicate RG thicknesses far in excess of ~20 m. As such, application of this alternate approach may significantly underestimate RG thicknesses.

Further regarding (b), here we assume estimated ice volume is 40–60% by volume^29–31,64^, enabling the calculation of lower (40%), average (50%), and upper (60%) estimates. Ice content within RGs is spatially heterogeneous. Therefore, the volumetric ice content varies strongly within a RG and between individual RGs. “The average volumetric ice content of rock glaciers is widely accepted to vary between approximately 40 per cent and 70 per cent…(Barsch, 1996: 40–60%; Burger *et al*. 50–70%)”^65^. This percentage array is consistent with field data from different climatic regions worldwide^22,31,66^. In this context, the assumption of an average 50% ice content is reasonable. Within the RGDB many studies assume RGs contain 40–60% ice content by volume; adoption of the same percentage array in this study enables inter-study comparative assessment. Furthermore, within the RGDB numerous studies classify RGs as intact, i.e. studies do not provide separate data for active and inactive RGs. Related to this, potential bias may be introduced by the typically lower ice contents of inactive RGs^65^. Additionally, information regarding RG genesis, i.e. permafrost origin or glacigenic origin, is predominantly absent within the RGDB despite strongly influencing ice content. Therefore, we acknowledge that due to the nature of the RGDB and the methodology, we cannot comprehensively account for regional specificities. Further research related to ice-thickness and ice content by volume is certainly needed.

## Conclusions

The significance of these results is fourfold. First, the systematic meta-analysis undertaken here has enabled the first near-global RGDB to be developed. This is based on the present state-of-knowledge of systematic RG inventory studies. Second, this work focuses on RGDB coverage and therefore enables identification of priority regions for systematic RG inventory studies, both at the RGI regional- and local- scales. Third, for the first time we present an assessment of water volume equivalents contained in the world’s observed RGs. These indicate that RG water stores are of potentially significant hydrological value (62.02 ± 12.40 Gt). In particular, our RGI regional approach indicates significant frozen water stores contained within RGs in arid and semi-arid high mountain systems facing potential future water scarcity (e.g., South America). Fourth, the methodology presented here enable an approximate comparative assessment of the ratios of RG to glacier water volume equivalents at RGI regional- and near-global-spatial scales. Finally, we acknowledge and discuss the uncertainty associated with the results presented here. These results represent a first-order approximation; uncertainty in the near-global RG water storage estimates is due to several factors, e.g., inherited errors within the RGDB, inherent flaws in the meta-analysis methodology, and RG thickness estimation, but here only errors associated with the assumption on RG ice content are quantified. Therefore, overall uncertainty is likely larger than that quantified here. Importantly, a full understanding of all inputs to the high mountain system hydrological is critical for effective water resource management to mitigate or adapt to the impacts of climate change – this includes RGs.

## Methods

### Rock glacier database (RGDB) collation

RG studies published prior to October 2017 were identified by means of journal search tools (ISI Web of Science, Scopus, ProQuest Dissertations and Theses), online databases (NSIDC), and direct communication with academics involved in RG research. We searched the ISI Web of Science for peer-reviewed journal papers published between 1900–2017 using topic searches for “rock glacier” OR “rockglacier” OR “rock glaciers” OR “rockglaciers”. Scopus was searched for peer-reviewed journal papers with ‘Document Type’ restricted to ‘Article’, ‘Conference Paper’, ‘Review’, and ‘Article in Press’ and no time-period restriction, also using the search terms “rock glacier” OR “rockglacier” OR “rock glaciers” OR “rockglaciers”. ProQuest Dissertations and Theses was searched for publications with full-text availability, using the search terms “rock glacier” OR “rockglacier” OR “rock glaciers” OR “rockglaciers”. Note that dissertations and theses with research outcomes already published as journal papers, were not included in the RGDB. Lastly, Google Scholar and NSIDC searches for “rock glacier” OR “rockglacier” OR “rock glaciers” OR “rockglaciers” were undertaken. Search results from ISI Web of Science and Scopus were categorised into (i) systematic inventory resources or (ii) not relevant.

Excluding duplicate studies, a total of 131 systematic RG inventory studies resulted from the systematic meta-analysis. So as to avoid duplicate RG data, i.e. overlapping study areas, 55 studies were excluded from the RGDB where more comprehensive and/or up-to-date inventories included the same RGs. This process was undertaken through Google Earth (version 7.1.5.1557, Google Inc., California, USA) and ArcGIS (version 10.3.1, ESRI, Redlands, CA, USA). Partially overlapping study areas were partially excluded. For example, data from Cremonese *et al*.^26^ (European Alps) was partially excluded where the study area overlapped that of Winkler *et al*.^40^ (Niedere Tauern Range, Austria).

The full RGDB structure required that the following fields be filled, where the data was available: (i) Source; (ii) Author(s) (including full citation); (iii) Study Location; (iv) Datasets Applied: (a) image dataset(s), (b) topographic dataset(s); (v) Inventory Validation: (a) Yes, (b) No, (c) NA (i.e. unknown); (vi) Number of Rock Glaciers: (a) total, (b) intact, (c) relict; (vii) Elevation (All, Intact, Relict): (a) mean(s), (b) minimum elevation at the front (MEF), (c) maximum elevation of the landform (MaxE); (viii) Area (All, Intact, Relict): (a) total, (b) mean(s); (ix) Length (All, Intact, Relict): (a) mean(s), (b) maximum(s); (x) Width (All, Intact, Relict): (a) mean(s), (b) maximum(s); (xi) Planform-shape (tongue-shaped, lobate, spatulate, or coalescent); (xii) Dominant Aspect(s); (xiii) Water Volume Equivalent (WVEQ); (xiv) Specific Density; (xv) Ratio of Rock Glacier WVEQ to Glacier WVEQ; (xvi) Additional Information. Note that where inventory data is missing but calculable (e.g., dataset[s] provided as supplementary information files, requires unit conversion etc.), we reflect updated values in blue font within the full RGDB.

Supplementary Fig. S1 and Supplementary Table S2 illustrate the 19 first-order regions that form the spatial structure of the RGIv4.0^32^. Further information is available from the Global Land Ice Measurements from Space (GLIMS) website (for access: http://www.glims.org/RGI/). In compiling the RGDB, a decision to merge consensus areas was taken for two key regions because the systematic RG inventory studies could not be split easily to account for regional differences. Here, we combined the RGIv4.0 regions: (i) ‘01’ (Alaska) and ‘02’ (Western Canada and US) to create a new dataset for “North America”; and (ii) ‘16’ (Low Latitudes) and ‘17’ (Southern Andes) to create a new dataset for “South America”, where there are high concentrations of both RGs and glaciers. Regarding (ii), sites in Central America, Africa, and Southeast Asia, which contained relatively insignificant proportions of RGs or glaciers, were grouped within the “South America” category. Systematic RG inventories resulting from the meta-analysis were similarly divided into the 17 regions (Supplementary Table S2).

### Estimating rock glacier hydrological stores

Estimations of RG water volume equivalent were calculated based upon assumed ice volumes stored within intact RGs. In order to place this work in the context of traditional glacier studies, the units of giga tons (Gt) are used. Here, T-A scaling relations, i.e. *H* = *cA*^*β*^ where mean RG thickness (H) is calculated as a function of surface area (A) and two scaling parameters (c and β), were applied. Scaling parameters derived from the empirical rule established by Brenning^28^ were used (equation [1]). RG volume was estimated through multiplication of (H) and (A). This approach has previously been applied in other studies^46,67,68^. Importantly, it should be noted that further research is needed to improve area-thickness relationships.

1 $$H=50\times [k{m}^{2(0.2)}]$$

By definition, RGs are ice-supersaturated accumulations of rock debris, and thus do not contain 100% ice. As such, ice content in RGs is spatially heterogeneous. Additionally, establishing RG genesis, i.e. permafrost origin or glacigenic origin, and subsequent depth and distribution of ice is challenging^69^. Consequently, estimation of ice volume and thus water volume equivalent proves difficult. The genesis of RGs remains contested; this controversy between supporters of the permafrost model (i.e. ice-cemented structure [permafrost origin]) versus those that support the glacier ice core model (i.e. glacier ice cored structure [glacigenic origin]) has previously been summarised and discussed in detail^25,70^. Note that discussion of RG genesis and evolution is beyond the scope of this study and is briefly highlighted here for completeness. Relatively few geophysical investigations of RG internal structure have previously been conducted. Those studies that exist often focus on quantifying ice presence opposed to ice content by volume. Therefore, here we assume estimated ice volume is 40–60% by volume^29–31,64^, enabling lower (40%), average (50%), and upper (60%) estimates to be calculated. Finally, water volume equivalent was calculated assuming an ice density conversion factor of 900 kg m^−3^.

Where complete RG inventories were available, RG surface area data were extracted for each individual feature for use in the abovementioned T-A relationship and subsequently water volume equivalent calculation. A three-step approach to determine RG volume was applied where inventory data was incomplete or unknown (Supplementary Fig. S2).

### Estimating glacier hydrological stores

Regarding glaciers, volume-area (V-A) scaling relations, i.e. *V* = *cA*^*γ*^ where glacier volume (V) is calculated as a function of surface area (A) and two scaling parameters (c and γ), are frequently used approaches for volume estimations^58^. Indeed, previously V-A approaches have been used in rock glacier-glacier comparative studies^46,68^. Furthermore, V-A approaches have been applied to global-scale volume estimations of glaciers and ice caps^71^. Reports indicate, however, the potential of V-A approaches to systematically overestimate ice volume, particularly for large and/or relatively steep glaciers (e.g., those within the Himalayan-Karakoram region^58^. Estimated ice volumes derived from ice-thickness distribution models, for instance the model of Huss and Farinotti^35^ (HF-model), generally yield comparatively lower results than V-A approaches^58^. Additionally, HF-model ice-thickness results have previously been validated, indicating good agreement with a comprehensive set of ice-thickness observations from almost all glacierized mountain ranges globally^34,35,58^. Direct validation cannot be undertaken for results derived from V-A relations^58^. Therefore, here we use the results of Huss and Hock^34^. For each glacier of the RGIv4.0, Huss and Hock^34^ calculated glacier volume and ice thickness distribution through the application of the HF-model^35^. Results within Huss and Hock^34^ were presented as SLE assuming an ice density of 900 km m^−3^ and an ocean area of 3.625 × 10^8^ km^2^. As such, conversion of SLE to ice volume was necessary. When converting from cubic kilometres to gigatons, we assumed that 1 Gt of nonporous ice equated to a volume of 1.091 km³ ^72^.

### Data availability

The datasets (RGDB) generated during and/or analysed during the current study are available in the supplementary information online.

## Electronic supplementary material

Supplementary Information

Dataset 1
