# Author Correction: Mountain rock glaciers contain globally significant water stores

**DOI:** 10.1038/s41598-021-00027-w

**Published:** 2021-10-19

**Authors:** D. B. Jones, S. Harrison, K. Anderson, R. A. Betts

**Affiliations:** 1 College of Life and Environmental Sciences, University of Exeter, Penryn Campus, Penryn, TR10 9EZ Cornwall UK; 2 Environment and Sustainability Institute, University of Exeter, Penryn Campus, Penryn, TR10 9EZ Cornwall UK; 3 College of Life and Environmental Sciences, University of Exeter, Streatham Campus, Exeter, EX4 4QE UK; 4 MetOffice, FitzRoy Road, Exeter, EX1 3PB Devon UK

Correction to: *Scientific Reports* 10.1038/s41598-018-21244-w, published online 12 February 2018

The original version of this Article contained errors.

Table 1 contained the incorrect values for the “South America” region. As a result, the values for “Near-Global” was incorrect. The correct and incorrect values appear below.

Incorrect:

**Table:** 

RGI region	No. studies (*n*)	Rock glaciers (*n*)			Rock glacier area			WVEQ
		Total	Intact	Relict	Total	Intact	Relict	
	(-)	(-)	(-)	(-)	(km^2^)	(km^2^)	(km^**2**^**)**	(Gt)
South America	17	28,665	16,117	12,548	3557.69	2307.60	1299.40	32.84 ± 6.57
**NEAR-GLOBAL**	**76**	**73,096**	**39,321**	**33,724**	**8879.79**	**5627.89**	**3436.06**	**83.72** ± **16.74**

Correct:

**Table:** 

RGI region	No. studies (*n*)	Rock glaciers (*n*)			Rock glacier area			WVEQ
		Total	Intact	Relict	Total	Intact	**Relict**	
	(-)	(-)	(-)	(-)	(km^2^)	(km^2^)	**(km**^**2**^**)**	**(Gt)**
South America	17	6991	4579	2412	976.51	725.01	251.66	11.14 ± 2.23
**NEAR-GLOBAL**	**76**	**51,422**	**27,783**	**23,588**	**6298.62**	**4045.30**	**2388.33**	**62.02 ± 12.40**

In addition, Table 2 contained the incorrect values for the “South America” region. As a result, the values for “Near-Global” was incorrect. The correct and incorrect values appear below.

Incorrect:

**Table:** 

RGI region	Rock glacier		Glacier		Ratio
	Area	WVEQ	Area	WVEQ	Rock glacier: glacier WVEQ
	(km^2^)	(Gt)	(km^2^)	(Gt)	
South America	3557.69	32.84	31,679.00	4,873.21	1:148
**NEAR-GLOBAL**	**8879.79**	**83.72**	**726,792.00**	**138,074.14**	**1:1,649**

Correct:

**Table:** 

RGI region	Rock glacier		Glacier		Ratio
	Area	WVEQ	Area	WVEQ	Rock glacier: glacier WVEQ
	(km^2^)	(Gt)	(km^2^)	(Gt)	
South America	976.51	11.14	31,679.00	4,873.21	1:437
**NEAR-GLOBAL**	**6298.62**	**62.02**	**726,792.00**	**138,074.14**	**1:2,226**

The original Article has been corrected.

