# Author Correction: Mountain rock glaciers contain globally significant water stores

**DOI:** 10.1038/s41598-021-02401-0

**Published:** 2021-12-01

**Authors:** D. B. Jones, S. Harrison, K. Anderson, R. A. Betts

**Affiliations:** 1 College of Life and Environmental Sciences, University of Exeter, Penryn Campus, Penryn, Cornwall, TR10 9EZ UK; 2 Environment and Sustainability Institute, University of Exeter, Penryn Campus, Penryn, Cornwall, TR10 9EZ UK; 3 College of Life and Environmental Sciences, University of Exeter, Streatham Campus, Exeter, EX4 4QE UK; 4 Met Office, FitzRoy Road, Exeter, Devon, EX1 3PB UK

Correction to: *Scientific Reports* 10.1038/s41598-018-21244-w, published online 12 February 2018

The original version of this Article contained errors.

In the Abstract,

“Here, we present the first near-global RG database (RGDB) through an analysis of current inventories and this contains > 73,000 RGs. Using the RGDB, we identify key data-deficient regions as research priorities (e.g., Central Asia). We provide the first approximation of near-global RG water volume equivalent and this is 83.72 ± 16.74 Gt. Excluding the Antarctic and Subantarctic, Greenland Periphery, and regions lacking data, we estimate a near-global RG to glacier water volume equivalent ratio of 1:456.”

now reads:

“Here, we present the first near-global RG database (RGDB) through an analysis of current inventories and this contains > 51,000 RGs. Using the RGDB, we identify key data-deficient regions as research priorities (e.g., Central Asia). We provide the first approximation of near-global RG water volume equivalent and this is 62.02 ± 12.40 Gt. Excluding the Antarctic and Subantarctic, Greenland Periphery, and regions lacking data, we estimate a near-global RG to glacier water volume equivalent ratio of 1:618.”

In the Results and Discussion section, under the subheading ‘Glacier- and rock glacier- hydrological value’,

“The RGDB presented here contains 73,096 RGs (intact = 39,321, relict = 33,724) covering an estimated area of ~ 8880 km^2^. From this, we present a first-order approximation of volumetric ice content contained within intact RGs. In total, we estimate that intact RGs contain a total ice volume of ~ 93 Gt assuming 50% ice content by volume. Therefore, intact RGs contain a total water volume equivalent of between 66.97 and 100.46 Gt, equivalent to ~ 68–102 trillion litres (Table 1), if a possible range of ice content between 40% and 60% is considered. Regionally, intact RGs located within South America (32.84 ± 6.57 Gt), South Asia East (19.48 ± 3.90 Gt), and North America (15.57 ± 3.12 Gt) likely contain the largest water stores. Conversely, water volume equivalents found within the Antarctic and Subantarctic, Greenland Periphery, New Zealand, and Scandinavia RGI regions are the smallest, with the upper estimate (i.e. 60% ice content by volume) containing < 0.88 Gt combined. Importantly, long-term RG water stores in arid and semi-arid regions are large (e.g., South America = 32.84 ± 6.57 Gt).”

now reads:

“The RGDB presented here contains 51,422 RGs (intact = 27,783, relict = 23,588) covering an estimated area of ~ 6,300 km^2^. From this, we present a first-order approximation of volumetric ice content contained within intact RGs. In total, we estimate that intact RGs contain a total ice volume of ~ 69 Gt assuming 50% ice content by volume. Therefore, intact RGs contain a total water volume equivalent of between 49.61 and 74.42 Gt, equivalent to ~ 54–81 trillion litres (Table 1), if a possible range of ice content between 40% and 60% is considered. Regionally, intact RGs located within South Asia East (19.48 ± 3.90 Gt), North America (15.57 ± 3.12 Gt) and South America (11.14 ± 2.23 Gt) likely contain the largest water stores. Conversely, water volume equivalents found within the Antarctic and Subantarctic, Greenland Periphery, New Zealand, and Scandinavia RGI regions are the smallest, with the upper estimate (i.e. 60% ice content by volume) containing < 0.88 Gt combined. Importantly, long-term RG water stores in arid and semi-arid regions are large (e.g., South America = 11.14 ± 2.23 Gt).”

Additionally,

“As a result, the total ratio of RG to glacier water volume equivalent is estimated to be 1:1,649 (Table 2). This implies that glaciers contain a store of water 1,649 larger than that of RGs at the near-global scale.”

now reads:

“As a result, the total ratio of RG to glacier water volume equivalent is estimated to be 1:2,226 (Table 2). This implies that glaciers contain a store of water 2,226 larger than that of RGs at the near-global scale.”

Furthermore,

“Excluding those RGI regions where no systematic RG inventory studies have been undertaken (i.e. Arctic Canada North, Arctic Canada South, Russian Arctic, and South Asia West), the estimated ratio of RG to glacier water volume equivalence is 1:1,098. For completeness we also excluded the Antarctic and Subantarctic and Greenland Periphery RGI regions, similar to other studies^45^, along with the aforementioned RGI regions where no systematic RG inventories have been undertaken. The resulting estimated ratio of RG to glacier water volume equivalence globally is 1:456.”

now reads:

“Excluding those RGI regions where no systematic RG inventory studies have been undertaken (i.e. Arctic Canada North, Arctic Canada South, Russian Arctic, and South Asia West), the estimated ratio of RG to glacier water volume equivalence is 1:1,482. For completeness we also excluded the Antarctic and Subantarctic and Greenland Periphery RGI regions, similar to other studies^45^, along with the aforementioned RGI regions where no systematic RG inventories have been undertaken. The resulting estimated ratio of RG to glacier water volume equivalence globally is 1:618.”

Lastly, under the subheading ‘Conclusions’,

“These indicate that RG water stores are of potentially significant hydrological value (83.72 ± 16.74 Gt).”

now reads:

“These indicate that RG water stores are of potentially significant hydrological value (62.02 ± 12.40 Gt).”

The original Article has been corrected.

