# Supplementary material for: Mountain rock glaciers contain globally significant water stores
Source: Sci Rep. 2018 Feb 12;8:2834. doi: 10.1038/s41598-018-21244-w (PMC5809490; doi:10.1038/s41598-018-21244-w)
Supplement: Supplementary file 1 — Supplementary Information [file 41598_2018_21244_MOESM1_ESM.pdf]

Mountain rock glaciers contain globally significant water stores

D. B. Jones,[1]* S. Harrison,[1] K. Anderson,[2] and R. A. Betts[3,4]

[1]College of Life and Environmental Sciences, University of Exeter, Penryn Campus, Penryn, Cornwall, TR10 9EZ, UK.
[2]Environment and Sustainability Institute, University of Exeter, Penryn Campus, Penryn, Cornwall, TR10 9EZ, UK.
[3]College of Life and Environmental Sciences, University of Exeter, Streatham Campus, Exeter, EX4 4QE, UK.
[4]Met Office, FitzRoy Road, Exeter, Devon, EX1 3PB, UK

Corresponding Author (*): Email: dj281@exeter.ac.uk

Supplementary Information

The Supplementary Information file includes:

- Supplementary Figures S1 and S2.
- Supplementary Tables S1 and S2.

Supplementary Figures

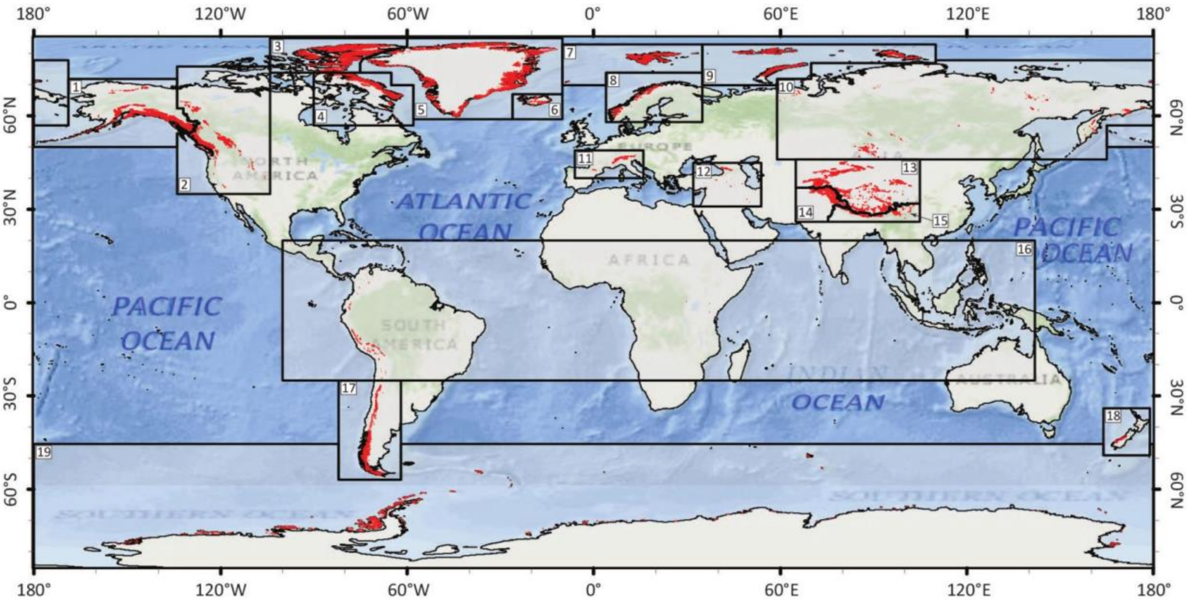

Supplementary Fig. S1. First-order regions of the RGIv4.0, with glaciers shown in red. RGI region numbers are summarised in Supplementary Table S2. Figure reprinted from Pfeffer et al.[33].

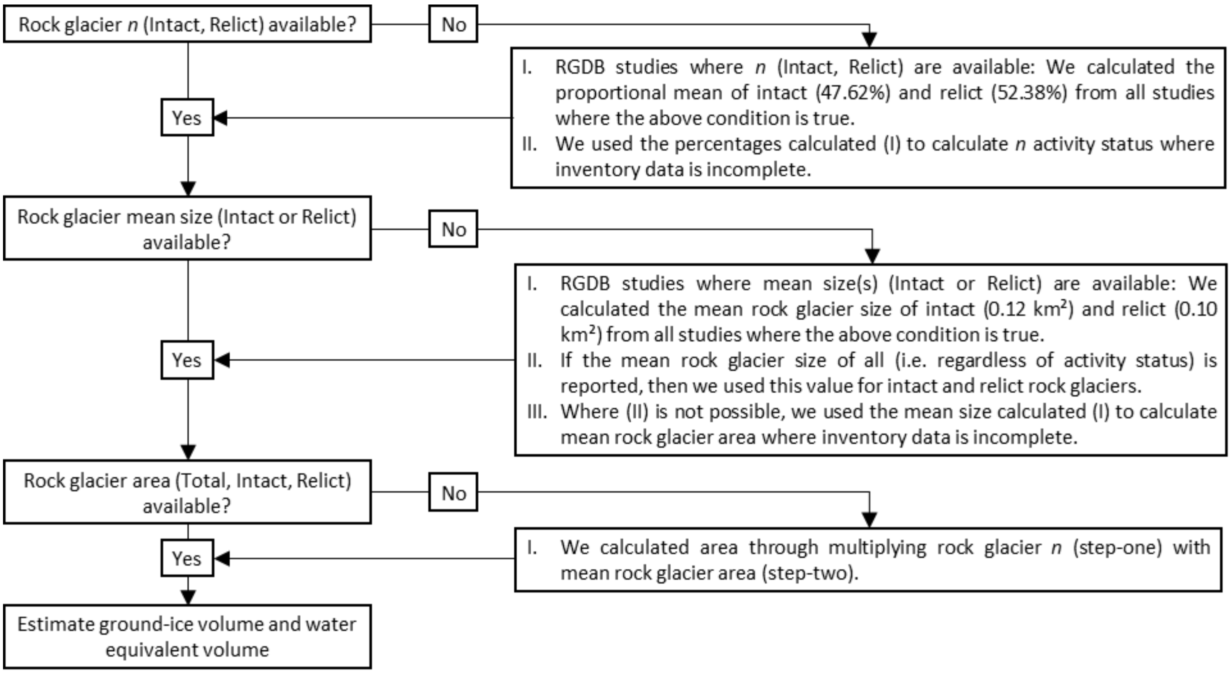

Supplementary Fig. S2. Workflow to calculate incomplete systematic RG inventory data, and subsequently ground-ice volume and water volume equivalent.

Supplementary Tables

Supplementary Table S1. Results of RGDB searches. Note that duplicate studies in ISI Web of Science and Scopus (*n* = 579) are excluded from the latter.

Source	*n*	Category	
		(I)	(II)
ISI Web of Science	799	70	729
Scopus	1023	14	430
ProQuest Dissertations and Theses	357	4	353
Google Scholar	26	26	-
NSIDC	13	13	-
Personal Communication	4	4	-

Supplementary Table S2. Glacier ice volume (Gt) is converted from the SLE data of Huss and Hock[34], assuming an ice density of 900 kg m^{-3}, an ocean area of 3.625 x 10^8 km^2, and that 1 Gt of nonporous ice equates to 1.091 km^3 [72]. 'Years' reflects the average satellite acquisition date for each glacier outline in the region (±1 standard deviation). First-order regions of the RGIv4.0 are reflected here. This table has been adapted from Huss and Hock[34].

RGI region		*n*	Area	SLE	Ice volume	Years
		(-)	(km^2)	(mm)	(Gt)	(-)
01	Alaska	26,944	86,715	45.28	16,716.57	2009 ± 2
02	Western Canada and US	15,215	14,559	2.47	911.88	2004 ± 5
	North America	42,159	101,274	47.75	17,628.45	-
03	Arctic Canada North	4538	104,873	67.02	24,742.59	1999 ± 0
04	Arctic Canada South	7347	40,894	19.70	7,272.89	2000 ± 6
05	Greenland Periphery	19,323	89,721	37.81	13,958.78	2001 ± 2
06	Iceland	568	11,060	8.13	3,001.45	2000 ± 1
07	Svalbard and Jan Mayen	1615	33,922	19.93	7,357.80	2007 ± 6
08	Scandinavia	2668	2851	0.36	132.91	2001 ± 2
09	Russian Arctic	1069	51,592	30.68	11,326.51	2002 ± 3
10	North Asia	4403	3430	0.40	147.67	1970 ± 19
11	Central Europe	3920	2063	0.28	103.37	2003 ± 5
12	Caucasus and Middle East	1386	1139	0.15	55.38	2000 ± 15
13	Central Asia	46,543	62,606	9.99	3,688.13	1970 ± 8
14	South Asia West	22,822	33,859	7.56	2,791.02	2000 ± 11
15	South Asia East	14,095	21,799	2.99	1,103.85	2000 ± 17
16	Low Latitudes	2863	2346	0.20	73.84	2002 ± 3
17	Southern Andes	16,046	29,333	13.00	4,799.37	2000 ± 0
	South America	18,909	31,679	13.20	4,873.21	-
18	New Zealand	3537	1162	0.15	55.38	1978 ± 0
19	Antarctic and Subantarctic	2752	132,867	107.90	39,834.76	1989 ± 15
**GLOBAL**		**197,654**	**726,792**	**374.00**	**138,074.14**	**-**
